# Surface Enhanced Raman Spectroscopy With Electrodeposited Copper Ultramicro-Wires With/Without Silver Nanostars Decoration

**DOI:** 10.3390/nano11020518

**Published:** 2021-02-18

**Authors:** Margherita Longoni, Maria Sole Zalaffi, Lavinia de Ferri, Angela Maria Stortini, Giulio Pojana, Paolo Ugo

**Affiliations:** 1 Department of Molecular Sciences and Nanosystems, University Ca’ Foscari of Venice, via Torino 155, 30172 Venice, Italy; margherita.longoni@unimi.it (M.L.); mariasole.zalaffi@unive.it (M.S.Z.); stortini@unive.it (A.M.S.); 2 Department of Chemistry, University of Milan, via C. Golgi 19, 20133 Milano, Italy; 3 Department of Philosophy and Cultural Heritage, University Ca’ Foscari of Venice, Dorsoduro 3484/d, 30123 Venice, Italy; lavinia.deferri@unive.it (L.d.F.); jp@unive.it (G.P.); 4 Department of Collection Management-Museum of Cultural History, University of Oslo, Kabelgata 34, 0580 Oslo, Norway

**Keywords:** copper, ultramicrowire, silver nanostars, template, electrochemical deposition, anodized aluminum oxide, track-etched polycarbonate, SERS, benzenethiol

## Abstract

The electrochemical preparation of arrays of copper ultramicrowires (CuUWs) by using porous membranes as templates is critically revisited, with the goal of obtaining cheap but efficient substrates for surface enhanced Raman spectroscopy (SERS). The role of the materials used for the electrodeposition is examined, comparing membranes of anodized aluminum oxide (AAO) vs. track-etched polycarbonate (PC) as well as copper vs. glassy carbon (GC) as electrode material. A voltammetric study performed on bare electrodes and potentiostatic tests on membrane coated electrodes allowed the optimization of the deposition parameters. The final arrays of CuUWs were obtained by chemical etching of the template, with NaOH for AAO and CH_2_Cl_2_ for PC. After total etching of the template, SERS spectra were recorded on CuUWs using benzenethiol as SERS probe with known spectral features. The CuUW substrates displayed good SERS properties, providing enhancement factor in the 10^3^–10^4^ range. Finally, it was demonstrated that higher Raman enhancement can be achieved when CuUWs are decorated with silver nanostars, supporting the formation of SERS active hot-spots at the bimetallic interface.

## 1. Introduction

Recent years have seen increased interest in the development of miniaturized analytical sensors based on arrays of ultramicro- and nanowires [1,2,3] which, thanks to their microscopic structure, allow to achieve significantly improved analytical performances, especially as far as sensitivity and possibility of miniaturization are concerned [4,5,6]. Here we will use the term nanowire (NW) and ultramicrowire (UW) for a cylindrical conductor with diameter ≤ 100 nm and in the 100–500 nm range, respectively. It is worth reminding that a micro- or nanowire is defined as any cylindrical conductor with aspect ratio (i.e., length/diameter) ≥ 20 [7]. Numerous examples of novel sensors based on arrays of NWs or UWs, which exploit electrochemical or optical transduction, have been presented and reviewed [1,2,3,8,9,10]. 

One of the most widely used methods for preparing arrays of microscopic wires is the membrane templated deposition [11,12,13,14]. Typically, anodized aluminum oxide (AAO) or track-etched polycarbonate (PC) membranes are used to this aim [12,13,14,15,16,17,18,19,20]. From a morphological viewpoint, both AAO and PC membrane exhibit monodisperse pores, with a diameter determined by the anodization potential for the former and by the etching time for the latter [14]. AAO membranes are characterized by very dense and orderly pores, arranged in a hexagonal pattern, but are rigid and fragile. Track-etched PC membranes present instead sparse and randomly arranged pores, but with the advantage of a greater flexibility.

The growth of the nanowires in the template can be obtained by chemical or electrochemical methods [12,13,14,15,16]. In particular, the electrochemical deposition of metal nanowires requires that one membrane side is in direct electronic contact with a conductive surface (electrode). Interestingly, the electrochemical deposition starts from the interface between the electrode surface and the electrolyte at the bottom of the pore, to develop progressively along the main axes, so that, by controlling the deposition time it is possible to control the wire length [21,22,23,24]. In order to maximize the aspect ratio of the templated wires, the electrochemical deposition should be stopped when the filling of the pores is almost complete, that is a few seconds before the metal deposit begins to develop on the external surface of the membrane. The obtained nanowires can be separated by the template by physical [25,26] or chemical etching [27,28,29]. Recently, arrays of copper wires, both copper nanowires (CuNWs) and ultramicro-wires (CuUWs), gained the interest of researchers thanks to their catalytic, electronic, and photo(electro)chemical properties, together with a significantly lower cost than noble metal equivalents [30,31,32].

Indeed, several reports have dealt with the preparation and electrochemical sensing application of CuNWs arrays by template synthesis both in AAO [15,30,31] and track-etched PC membranes [22,23,33,34]. In the first part of the present work, we have revisited and compared the role of some parameters that can influence the electrochemical deposition of copper wires such as: (i) the nature of the template (AAO vs. PC); (ii) the material of the substrate electrode (copper vs. glassy carbon); and (iii) the concentration of Cu^2+^ ions in the electrolyte. In particular, we focused on arrays of CuUWs whose preparation can be easily performed in any chemical laboratory.

The obtained CuUWs have been examined with respect to their application as substrates for Surface Enhanced Raman Spectroscopy (SERS). SERS is a spectroscopic analytical technique, which takes advantage of the dramatic enhancement of the Raman signal produced by the interaction of the molecule under study with nanostructured metal surfaces [35,36,37,38]. This allows the sensitive detection of the analyte even in complex samples such as living cells [39,40]. Recent researches have demonstrated that membrane templated nanowires are highly effective in producing SERS effects [41,42,43,44,45]. but only few studies have examined the generation of SERS effects with copper wires of microscopic dimension [46,47,48], and no reports have examined the possibility to exploit membrane-templated CuNWs or CuUWs to this aim. Recently it was proven both experimentally and theoretically that decorating metal nanowires (in particular AuNWs) with silver anisotropic nanostructures, such as silver nanostars (AgNSs), further increases the SERS effect already produced by the nanowires [45]. Theoretical calculations demonstrated that this is related to the generation of SERS active hot spots at the interface between nanostars and nanowires. All these observations prompted us to study the possible extension of similar SERS effects to arrays of CuUW arrays, both before and after decoration with AgNSs.

## 2. Materials and Methods

### 2.1. Chemicals and Materials

All chemicals were of analytical grade and used without further purification. Solutions were prepared using double distilled water (18.2 MΩ cm^−1^). 

Nafion^®^ 117, 5% *w*/*v* hydroalcolic solution (Sigma-Aldrich) was diluted to 1% with methanol before use. 

The Cu deposition was typically performed at room temperature in electrolyte solutions of CuSO_4_ in 10^−2^ M H_2_SO_4_ and 2 M Na_2_SO_4_ [23]. 

The membranes used as templates were: (i) track-etched polycarbonate SPI-Pore membranes by SPI-Supplies (West Chester, PA, USA): pore diameter 400 nm, thickness 10 µm, pore density 1 × 10^8^ pores cm^−2^, treated by the producer with polyvinylpyrrolidone as wetting agent; (ii) anodized aluminum oxide AAO wafer membranes by InRedox (Longmont, CO, USA): pore diameter 200 ± 22 nm, thickness 50 ± 10 µm, pore density 5.7 × 10^8^ pores cm^−2^. 

**Figure 1:**
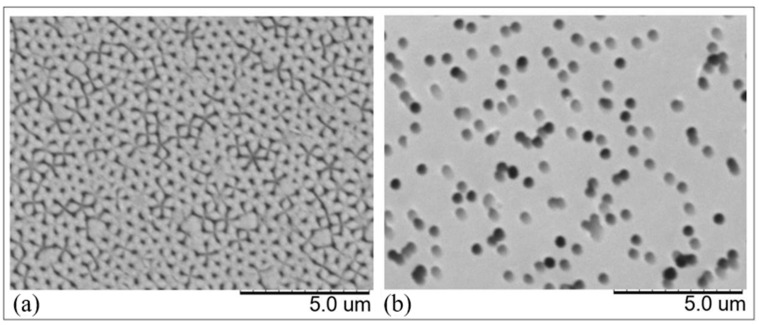
Scanning electron microscopy images of the surface of the membranes here used as templates: (**a**) anodized aluminum oxide; (**b**) track-etched polycarbonate.

Figure 1 shows the SEM images of the surface of the two AAO and PC membranes applied here. 

### 2.2. Electrochemical Methods

Electrochemical measurements were performed with a CHI1000 workstation (CH Instruments, Austin, TX, USA). Cyclic voltammetry (CV) was performed at room temperature using a three-electrode cell set up. The working electrode was a glassy carbon disk (GC) or a Cu disk electrode (diameter of 5 and 3 mm, respectively), mirror-polished with graded alumina (1, 3 and 0.5 µm), ultrasonicated and carefully rinsed with water before use. A Pt coil was used as a counter electrode. In order to prevent the presence of chloride ions, which can alter the reduction mechanism of copper [49], a copper plate was used as a pseudo-reference electrode, hereafter indicated as Cu(pseudo ref). 

**Figure 2:**
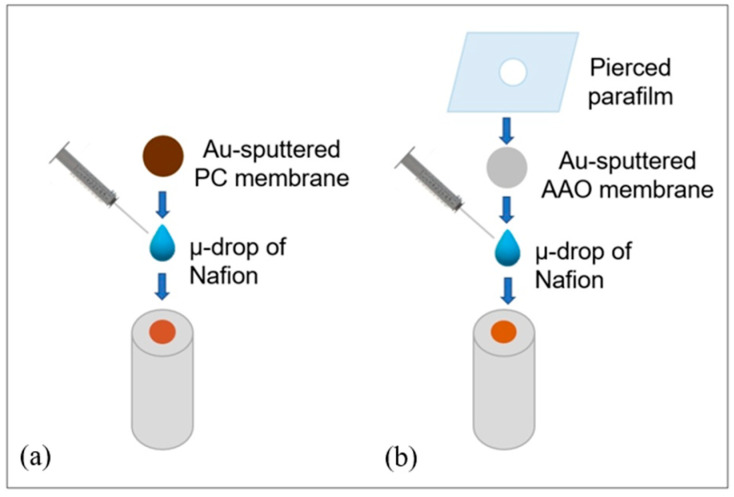
Scheme illustrating the method used to fix the template membrane on the working electrode for the case of: (**a**) track-etched polycarbonate; (**b**) anodized aluminum oxide.

All membrane templated electrochemical depositions were performed at room temperature operating at a constant potential, using a conventional single-compartment cell equipped with a copper plate counter electrode and a Cu(pseudo ref). The working electrodes were GC or Cu disk electrodes, described above, coated with the AAO or PC templating membrane by using the procedure schematized in Figure 2 and described in Section 2.3 and Section 2.4.

### 2.3. CuUWs by Polycarbonate Membrane

Copper ultramicrowires deposition was carried out on PC membranes with 400 nm pore diameter. To improve electrical contact with the supporting electrode, one side of the template membrane was sputtered with a thin layer of gold (average thickness 75 nm). In order to improve the adhesion between PC and the metal substrate, 10 µL of 1% *w*/*v* Nafion solution were used as ionically conductive glue [32]. The electrochemical deposition was carried out in 0.30 M CuSO_4_, 2 M Na_2_SO_4_, 10^−2^ M H_2_SO_4_ electrolyte solution. After the deposition, the PC template was removed by chemical etching with pure dichloromethane for one minute.

### 2.4. CuUWs by Alumina Membrane

The CuUWs deposition was carried out on AAO wafer membranes with pores of 200 nm diameter. As for the PC membrane, pre-sputtering with a thin gold layer was performed. In order to improve adhesion, in addition to using the microvolume of Nafion solution, the template was fixed on its outer border with Parafilm (Figure 2b). After the deposition in 0.30 M CuSO_4_, 2 M Na_2_SO_4_, 10^−2^ M H_2_SO_4_ solution, the AAO template was removed by chemical etching in 2 M NaOH for 5 min.

### 2.5. Preparation of Silver Nanostars

Colloidal dispersions of silver nanostars (AgNSs) were prepared using the one-pot method previously described [45,50,51] where hydroxylamine, citrate, and NaOH were used as reducing and shape directioning agents. 

**Figure 3:**
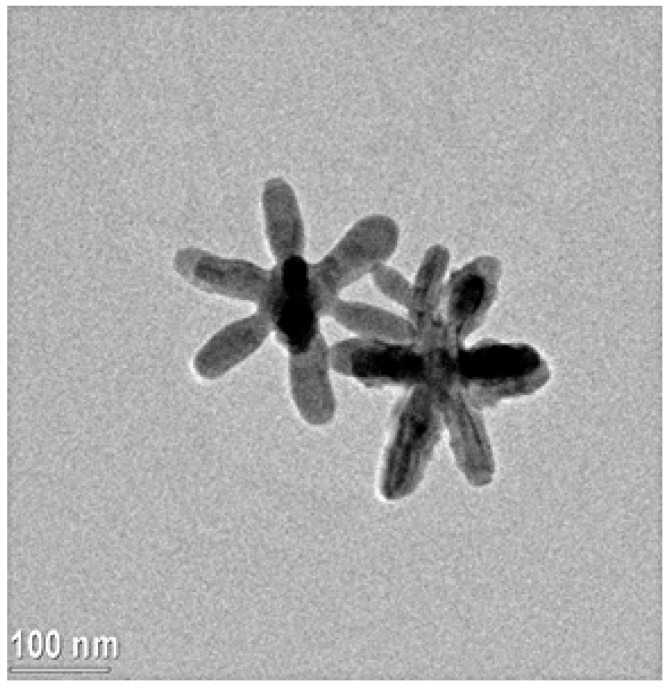
Transmission electron microscopy image of the silver nanostars.

Briefly, 0.5 mL of 0.05 M NaOH and 0.5 mL of hydroxylamine (0.18% *w*/*v*) solutions were mixed under stirring for one minute. Then, 9 mL of a 10^−3^ M AgNO_3_ solution were added with stirring for further 5 min. Afterwards, 100 µL of a 90.045 M citrate solution were dropped in the flask, continuing stirring for approximately 15 min, i.e., until it developed a dark green color. Then the flask was stored in the dark at room temperature to complete the growth of the star-shaped NPs, which took approximately 48 h. The suspension was concentrated before use as described in ref. [45]. Figure 3 shows the TEM image of the here-obtained nanostars.

### 2.6. Samples for SERS Analysis

For SERS measurements, benzenethiol (BT) was used as Raman probe with known spectral features. Before SERS analyses, the structures under study were incubated overnight in a 10^−3^ M BT solution in ethanol, followed by gentle washing with pure ethanol.

The structures used as SERS substrates were: (i) flat copper plate, used as reference material; (ii) CuUWs on GC; (iii) CuUWs decorated with AgNSs (AgNS@CuUW). The preparation of the final samples for SERS analyses are detailed below:

(i) Metallic copper plate (approximately, 1 cm × 0.8 cm): the metallic surface was mirror polished with fine grain emery paper and graded alumina powder (1, 3 and 0.5 µm granulometry), carefully rinsed with water just before use.

(ii) CuUWs: the arrays were grown on GC using PC as the templating membrane (see above). 

(iii) AgNS@CuUW: when required, the above CuUWs arrays were decorated with AgNSs by applying the procedure previously reported for similar nanostructures, but supported on gold nanowires. Briefly, the procedure included: (i) overnight immersion of the CuUWs in 10^−2^ M cysteamine solution in water; (ii) overnight immersion in a colloidal dispersion of silver nanostars (AgNSs). Careful washing with deionized water was performed between each step. Cysteamine was used to bridge the nanostars onto the ultramicrowires [45].

### 2.7. SERS Measurements

SERS analyses were performed using a B&WTek (Newark, DE, USA) i-Raman 785S spectrometer equipped with a diode laser operating at 785 nm. The maximum power was 300 mW and the nominal spectral resolution 4.5 cm^−1^. The spectrometer is coupled with BAC151B (B&W Tek, Inc.) microscope through optical fibers (1.5m length); a 20× objective was used to collect spectra in the 175–3000 cm^−1^ spectral range. with a typical integration time of 20 s. Spectra were acquired using the BWspec4 software; the post-processing of the data was performed with the OriginLab software.

### 2.8. Electron Microscopy 

Scanning Electron Microscopy (SEM) and energy dispersive spectroscopy (EDS) analysis were performed using a TM3000 Hitachi tabletop scanning electron microscope, coupled with an X-ray microanalysis system (SwiftED3000); conditions for recording the EDS spectra were: acquisition time 30.0 s; process time 5 s; accelerating voltage 15 kV. 

Transmission electron microscopy (TEM) was performed using a JEOL 3010 (0.17 nm point-to-point resolution at Scherzer defocus), operating at 300 kV, equipped with a Gatan slow-scan CCD camera (model 794).

## 3. Results and Discussion

### 3.1. Voltammetric Study of Cu^2+^ Reduction

In order to find the best conditions for the electrochemical deposition of copper, preliminary analyses were performed by cyclic voltammetry in CuSO_4_ solutions, with 2 M Na_2_SO_4_, 10^−2^ M H_2_SO_4_ as the supporting electrolyte [23]. Preliminary tests indicated that the CV patterns remained substantially unchanged varying both Na_2_SO_4_ and H_2_SO_4_ concentrations in the 0.5–2 M and 5 × 10^−3^–1 × 10^−2^ M range, respectively.

**Figure 4:**
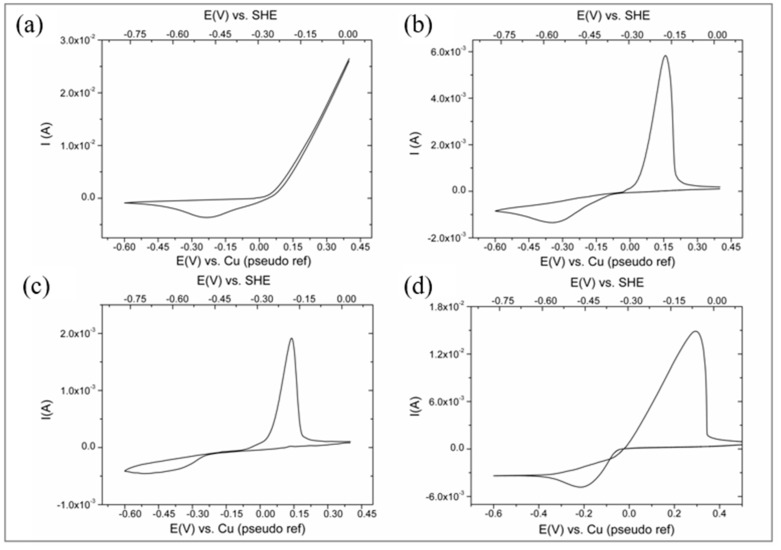
Cyclic voltammograms recorded at 40 mV/s in 0.01 M H_2_SO_4_, 2 M Na_2_SO_4_ solution: (**a**) with Cu working electrode in the presence of 0.05 M CuSO_4_; (**b**–**d**) with GC working electrode in the presence of 0.05 M, 0.01 M and 0.3 M CuSO_4_, respectively.

Figure 4 compares the cyclic voltammograms, recorded at 40 mV/s using a copper working electrode (A) and a GC-disk working electrode (B). On the copper working electrode, a cathodic peak was detected at around −0.230 V vs. Cu(pseudo ref) in the CV cathodic branch, while the anodic branch was characterized by an almost linear growth of the current at potentials higher than +0.02 V. The reduction peak was due to the two-electron reduction of Cu^2+^ ions, which diffused from the bulk solution to the surface of the electrode. The anodic current increased without creating a peak because it corresponded to the oxidation of both the metallic copper deposited during the cathodic branch of the CV and the metallic copper that constitutes the electrode itself. 

In the case of glassy carbon electrode (Figure 4b), the CV cathodic branch presented a peak at −0.35 V vs. Cu (pseudo ref), while the anodic one showed a symmetric peak at about +0.140 V vs. Cu(pseudo ref), showing the shape typical for a cathodic deposition and anodic stripping related to the two electron process:

Cu^2+^ + 2e^−^ ⇆ Cu^0^ (1)

The reduction peak of Cu^2+^ to Cu^0^ was shifted to slightly more negative potentials on GC with respect to copper. This is because the deposition of copper on GC is more energy demanding than the deposition of copper on copper, the first process being affected by a higher overpotential because of the energy required to form the first metallic nuclei on a foreign substrate (i.e., glassy carbon) [52,53].

Focusing on the deposition of Cu on GC, Figure 4c,d shows the CV recorded at different CuSO_4_ concentrations, namely 0.01 M and 0.3 M. 

It is worth reminding that the potential of the Cu(pseudo ref) depends on CuSO_4_ concentration. This is not a problem when operating always at the same Cu^2+^ concentration, but this dependence must be taken into account when operating with different Cu^2+^ concentrations. The Cu(pseudo ref) electrode behaves indeed as a 1st kind electrode whose potential [53] is given by:

E_Cu(pseudo ref)_ = E + 0.059/2 log [Cu^2+^] = 0.340 + 0.059/2 log [Cu^2+^] (V vs. SHE) (2)

where SHE is the standard hydrogen electrode.

It can be easily calculated that, in 1 × 10^−2^, 1 × 10^−2^ and 3 × 10^−1^ M CuSO_4_ solutions the potential of the Cu-pseudo is 0.280, 0.300, 0.320 V vs. SHE, respectively. 

Therefore, in order to perform a correct evaluation, in Figure 4, potential values were plotted also with respect to the ideal SHE reference electrode (see upper X-axis).

Table 1 reports relevant voltammetric parameters obtained from the above CVs as well as from those recorded changing the scan rate from 10 to 80 mV s. 

**Figure 5:**
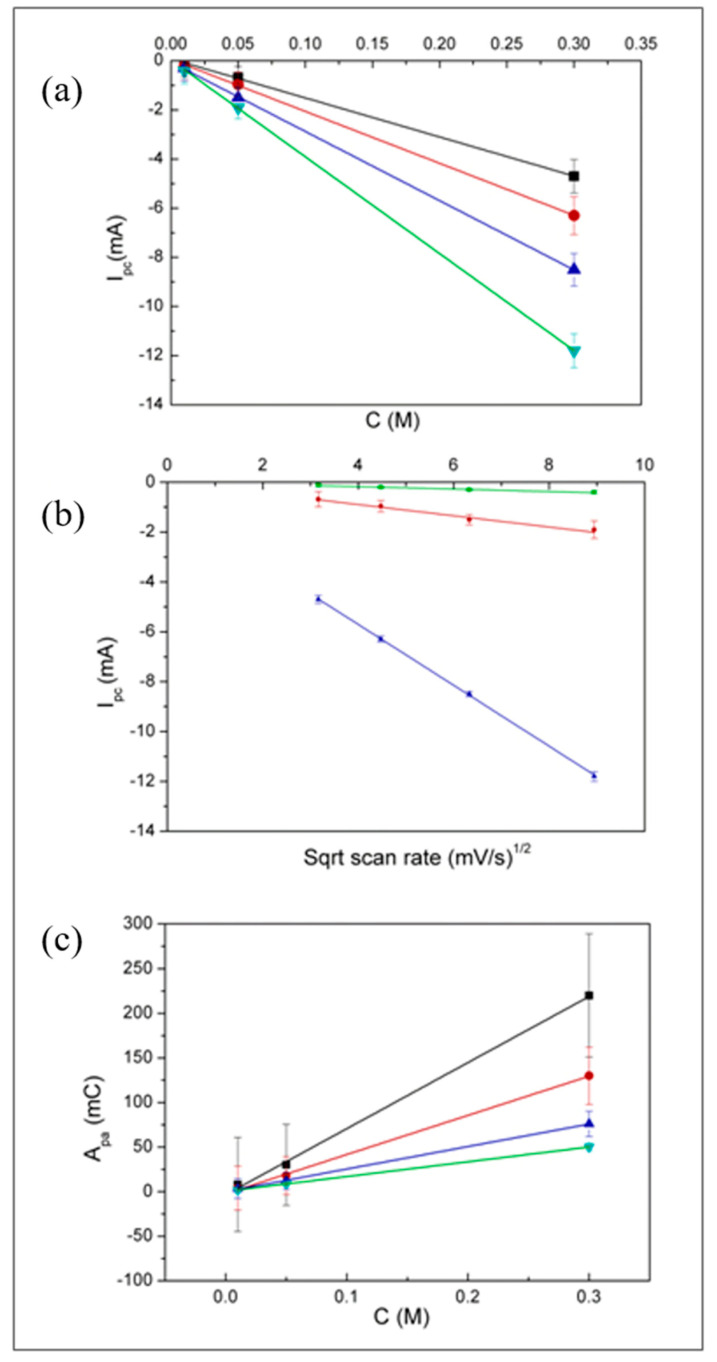
Plots obtained from data in Table 1, showing the dependence of: (**a**) cathodic peak current vs. CuSO_4_ concentration, at scan rate: 10 (black); 20 (red); 40 (blue); 80 mV/s (green); (**b**) cathodic peak current vs. square root of the scan rate, at [Cu^2+^]: 0.01 (green); 0.05 (red); 0.3 M (blue); (**c**) electric charge measured by integration of the anodic peak vs. CuSO_4_ concentration at scan rate: 10 (black); 20 (red); 40 (blue); 80 mV/s (green).

From these data, the plots shown in Figure 5 were obtained. Figure 5a shows that the cathodic peak current scales linearly with the Cu^2+^ concentration. The linear dependence of I_pc_ on the square root of the scan rate (see Figure 5b) indicates that, in the electrolyte here used, the reduction of Cu^2+^ is diffusion controlled [53], with no relevant effects of possible migration effects even at the highest Cu^2+^ concentration studied here, that is 0.30 M. 

The anodic peak current showed a more complex dependence both on Cu^2+^ concentration and scan rate; instead, as expected for a stripping peak, the charge, measured by integrating the anodic peak, scales linearly with the Cu^2+^ concentration. 

Data reported in Table 1 indicate that the cathodic peak potential shifts anodically with increasing the Cu^2+^ concentration, as expected for a reduction process related to the formation of a metal phase [54,55,56]:

From an electrodeposition viewpoint, this evidence indicates that it is more convenient to operate at relatively high Cu^2+^ solution concentrations (i.e., 0.30 M), where the reduction starts to occur at less-cathodic potential values. 

### 3.2. Optimization of CuUWs Deposition in PC Membranes

On the basis of the CV evidence, we examined the deposition of CuUWs on Cu and GC working electrodes coated with PC or AAO nanoporous membranes. 

#### 3.2.1. Template Deposition of CuUWs in Polycarbonate Membranes

On the basis of the CV results, the deposition was performed under potentiostatic control at −0.4 V vs. Cu(pseudo ref), using 0.3 M CuSO_4_, 2 M NaSO_4_, 10^−2^ M H_2_SO_4_ electrolyte, using a GC or Cu working electrode, coated with the PC membrane, as described in Section 2.3.

A key point for the successful growth of CuUW is the choice of an optimal deposition time. 

**Figure 6:**
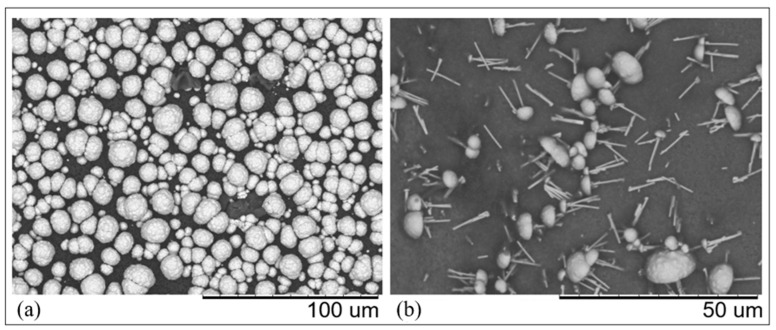
SEM images of the copper deposit obtained using track-etched PC templates onto a GC: (**a**) surface before etching; (**b**) mushroom-like nanostructures after etching in CH_2_Cl_2_ for 1 min. Deposition at −0.400 V vs. Cu(pseudo ref) for 600 s; etching in CH_2_Cl_2_ for 1 min.

Figure 6 shows the SEM image of the deposit obtained using a deposition time of 600s. When using such a deposition time, after filling the pores, copper is also deposited on the outer mouth of the pore, forming mushroom-like structures. On the other hand, other preliminary experiments indicated that deposition times of the order of 60 s or lower are too short to provide any satisfactory copper deposition inside the pores. Finally, it was found that the best time necessary to deposit continuous CuUWs in track-etched polycarbonate is in the 120–240 s range.

**Figure 7:**
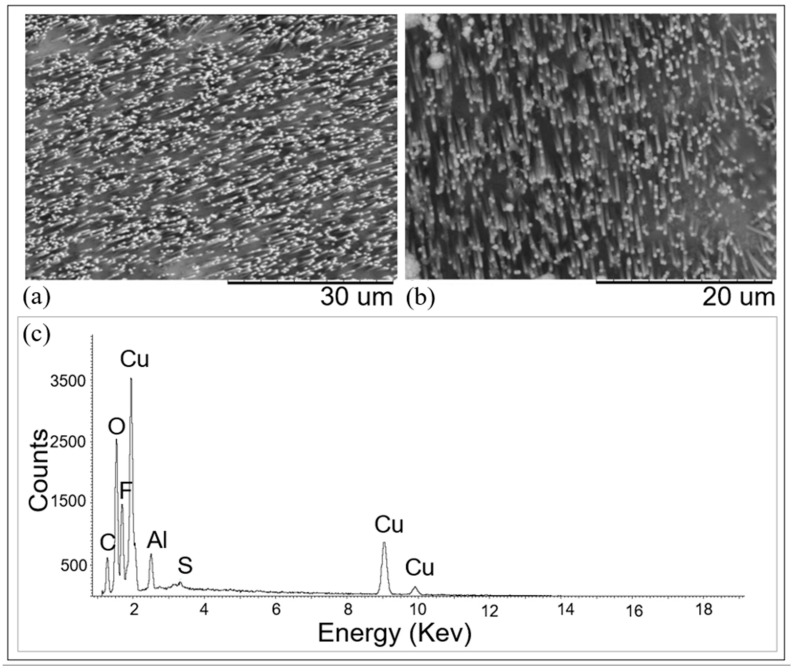
SEM images of CuUWs obtained using track-etched PC templates onto: (**a**) GC working electrode; (**b**) Cu working electrode. Deposition at −0.400 V vs. Cu(pseudo ref) for for 140 s (**a**) and 180 s (**b**); etching in CH_2_Cl_2_ for 1 min. (**c**) EDS spectrum recorded on the CuUWs obtained after template deposition (**a**).

Figure 7a shows the SEM image of the copper wires obtained by applying −0.4 V vs. Cu(pseudo ref) for 140 s to a GC working electrode coated by the PC template, and following etching of the template with CH_2_Cl_2_. The main EDS signals shown in Figure 7c confirmed the successful deposition of copper. Signals from other elements were also detected, in particular carbon, oxygen, sulphur, and fluorine, that could be attributed to residual traces of polycarbonate and Nafion.

Comparable results were obtained at the Cu working electrode; however, using 180 s as the deposition time, always at −0.4 V vs. Cu(pseudo ref).

As shown by data in Figure 7 and Figure S1 (in Supplementary Material), the length of the obtained wires was 10.0–10.7 µm, while their thickness was 350–410 nm, providing an aspect ratio between 24 and 30.

#### 3.2.2. Template Deposition of CuUWs in AAO Membranes

For the case of alumina membranes, no positive results were achieved when using the GC working electrode. This seems related to poor contact (or adhesion) between GC and AAO; the former is indeed hydrophobic and the latter hydrophilic. On the other hand, good results were obtained with the Cu working electrode, thanks to a good match between these two hydrophilic materials.

Similarly to what is described above for PC, preliminary tests allowed to determine the best conditions to deposit copper ultramicrowires in AAO templates on a Cu working electrode, which resulted in 300 s at −0.250 V vs. Cu(pseudo ref).

**Figure 8:**
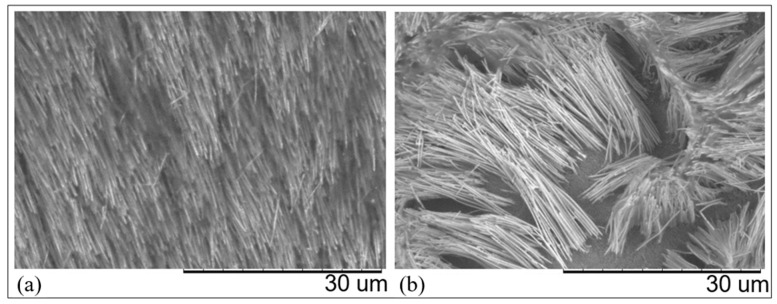
SEM images of: (**a**) self-standing, (**b**) collapsed CuUWs prepared by using a AAO template onto a Cu working electrode. Deposition at −0.250 V vs. Cu(pseudo ref) for 300 s; etching in 2 M NaOH for 5 min.

A longer deposition time was used for the AAO membranes because of their higher thickness. After the deposition, the membranes were etched in 2 M NaOH for 5 min. The SEM images reported in Figure 8 confirmed the successful growth of CuUWs, which show a length around 30 µm and an average thickness of about 250 nm (see also Figure S2 in Supplementary Material), which means an aspect ratio of around 120.

However, as shown by Figure 8b, the total etching of the template can cause the collapse of the wires, probably because of their excessive length and mechanical weakness of their basis. A similar effect was reported by Du et al. [43] for the case of silver nanowires in AAO, which collapsed after the total etching of the template, while they stayed stable when only partial etching of the membrane was operated.

In summary, the above results indicate that the growth of CuUWs within polycarbonate membranes occurs successfully on both glassy carbon and copper working electrodes, obtaining self-standing CuUW wires after the etching of the template. 

On the other hand, the growth of CuUWs on AAO was successful only on copper substrates; in this case, very high aspect ratio wires can be produced, however the obtained structures are much less robust, tending to collapse after the total etching of the membrane.

### 3.3. SERS Detection of Benzenethiol on CuUWs

In order to test the Raman enhancement effect produced by these copper-based SERS substrates, benzenethiol (BT) [50,51,52,53,54,55,56,57,58,59] was chosen as reference Raman probe. A copper plate, named hereafter macro-copper, was employed as control. 

Preliminary SERS tests indicated that scarcely reproducible spectra were obtained with the arrays of copper wires prepared in AAO templates. This has been attributed to the above described collapsibility of these wires, similarly to what was previously observed by Du et al. [43] for the case of silver nanowires prepared in AAO, which, once collapsed, became SERS inactive [43]. For this reason, the SERS studies reported below refer only to the more robust arrays of CuUWs obtained with a PC template.

**Figure 9:**
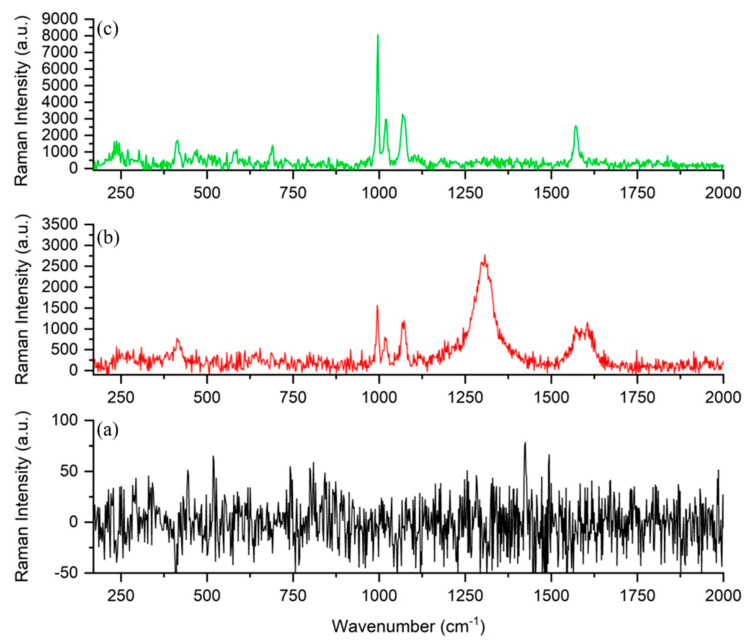
SERS spectra of 10^−3^ M BT recorded on (**a**) macro-copper, (**b**) CuUWs, and (**c**) AgNSs@CuUWs (λ_ex_= 785 nm, P = 15 mW, t = 3 × 20 s).

Figure 9 shows the spectra recorded when analyzing BT adsorbed on different substrates from a 10^−3^ M solution. On macro-copper (see Figure 9a), no BT signal was detected. Instead, when CuUWs from PC were employed as the substrate, the typical bands of BT were observed (Figure 9b), indicating the effectiviness of CuUW in producing a detectable enhancement of Raman signals. In particular, the collected spectra displayed three main bands, namely at 996 cm^−1^, 1022 cm^−1^, and 1072 cm^−1^, with the appearance of a weaker band at 466 cm^−1^ [57,58,59,60,61]. The detailed attribution of these, as well as the other BT Raman bands, observed in the collected spectra are reported in Table S1, in Supplementary Material. As can be observed, for some features, more than one possible attribution can be found in the literature. Different authors, provided, in fact, different attributions to different vibrational modes and Table S1 shows the most possible updated overview of given interpretations.

Interestingly, in spectrum (b), the detection of the BT peak expected near 1580 cm^−1^ [59,60] was indeed hindered by the overlap with two broad bands at 1305 and 1590 cm^−1^. These two bands resemble the Raman bands, named “D” (1360 cm^−1^) and “G” (1582 cm^−1^), typical of carbon microstructures, in particular of glassy carbon [57,62,63]. Here, their detection was attributed to the GC substrate on which the CuUW structure was grown. 

It was recently demonstrated that the combination of arrays of AuNWs with AgNS can bring higher SERS enhancement with respect to arrays of NWs alone, generating an increase in number and efficiency of the SERS-active hot-spots [43]. Therefore, here we tested whether the decoration of CuUWs with AgNSs could provide a similar effect. The spectrum in Figure 9c, recorded with AgNS@CuUW, displayed more intense peaks than spectrum (b). Note that the interference bands by GC became negligible, and the much more intense spectrum of the analyte is now well resolved from the background, thanks to the stronger enhancement characteristics of the bimetallic nano-system. 

In particular, the peak of BT at 1570 cm^−1^ emerged with practically no interference by the GC band at 1550–1600 cm^−1^. These results confirm the capability of AgNS to produce high SERS enhancements when combined with metal wires of nanoscopic dimension, as a consequence of the generation of highly efficient hot-spots where the stars are in contact the wires, as already demonstrated for the case of gold nanowires decorated with AgNSs [45].

With the aim of quantifying the SERS effect, enhancement factor (EF) values were estimated from the spectra collected on CuUWs and AgNS@CuUW [45,59] by using the area of the peak recorded at 1022 cm^−1^. The peak area values used in the calculation were the average peak area measured and averaged from three spectra recorded on three different substrate samples. The relative standard deviation (RSD) values of such measurements resulted in 17.5% and 13.5% for CuUW and AgNS@CuUW, respectively. For EF evaluation, at first, the approximate surface areas of CuUW, AgNSs, and relevant hierarchical structures were calculated, estimating geometrical and morphological parameters (namely, geometry, and spatial distribution of the particles) from SEM data [45]. The calculated values are listed in Table 2. Taking into account the laser spot diameter, the surface area of the nanostructures and the benzenethiol molecular area, i.e., 0.22 nm^2^/molecule [59,64], the number of molecules excited by the laser beam could be estimated. Enhancements factors (EF) were evaluated with respect to Raman spectra recorded in pure BT liquid, by using Equation (3) [65]:

EF = (*I*_SERS_/*N*_SERS_)/(*I*_Raman_/*N*_Raman_) (3)

where *I*_SERS_ (or *I*_Raman_) refers to the band integral, normalized by the laser power and the exposure time used for the acquisition, while *N*_SERS_ (or *N*_Raman_) refers to the number of BT molecules sampled within the scattering volume. Both *I*_Raman_ and *N*_Raman_ values were obtained by Raman measurements performed on pure BT solution [45], taken as reference for the EF calculation.

Estimated enhancement factors were 0.7 × 10^4^ and 1.6 × 10^4^ for CuUW and AgNS@CuUW, respectively. These values are similar to the 1.1 × 10^4^ EF value reported by Yang H-J et al. [47] for the case of 4-mercaptobenzoic acid adsorbed on (self-seeded) copper nanowires with 75 nm diameter and 18 µm length. With respect to BT adsorbed on AuNWs and AgNS@AuNW structures [45], the values obtained in this investigation are approximately one order of magnitude lower, indicating a lower efficiency of copper with respect to gold. From a practical viewpoint, the results here reported indicate that SERS effects attainable with CuUWs are still remarkable, supporting the applicability of cheaper than gold CuUWs for SERS measurements; if a greater sensitivity is requested, decoration with AgNS can be used. 

## 4. Conclusions

The results achieved in this work provide evidence that the growth of copper ultramicrowires in PC membranes occurs successfully both on copper and glassy carbon substrate electrodes, while for the case of AAO membranes, good results can be obtained only on copper electrodes, probably because of adhesion problems between AAO and GC. The choice of optimized deposition time and potential allow the preparation of ordered arrays of CuUWs with high aspect ratio both from AAO and PC; however, the nanostructures obtained from the PC template are mechanically more robust, probably because residual traces of PC left after the etching help in keeping a solid anchoring of the ultramicrowires on the substrate.

The developed CuUW substrates display good SERS enhancement properties for adsorbed benzenthiol, which are further improved after decoration with AgNSs. The sensitivity with CuUW and AgNS@CuUW substrates is slightly lower than that with AuNWs or AgNS@AuNW. Further focused studies are required to understand if such differences are related to the nature or morphology of these CuUW or are rather due to differences in the interaction of BT with CuUW vs. AuNWs. Anyhow, the results here presented indicate that CuUW structures can be useful for studying Raman active molecules, in particular for obtaining mechanistic information on catalytic processes where copper is involved, or when efficient but cheap SERS substrates are desired.

## Figures and Tables

**Table 1:** Voltammetric parameters measured from the cyclic voltammograms recorded using the experimental conditions indicated in Figure 4.

[CuSO_4_] (M)	Scan Rate (mV/s)	E_pc_ vs. Cu (V)	E_pc_ vs. SHE (V)	I_pc_ (mA)	E_pa_ vs. Cu (V)	E_pa_ vs. SHE (V)	I_pa_ (mA)	A_pa_ (mC)
0.01	10	−0.290	−0.010	−0.12	0.130	0.410	1.10	8.0
	20	−0.350	−0.070	−0.20	0.135	0.415	1.48	4.0
	40	−0.380	−0.100	−0.30	0.138	0.418	1.90	3.5
	80	−0.535	−0.255	−0.40	0.140	0.420	2.10	1.7
0.05	10	−0.280	−0.020	−0.68	0.132	0.432	3.50	30
	20	−0.310	−0.010	−0.96	0.140	0.439	4.58	18.0
	40	−0.340	−0.040	−1.50	0.156	0.456	5.80	12.0
	80	−0.370	−0.070	−1.90	0.167	0.467	6.90	8.8
0.30	10	−0.215	0.105	−4.7	0.285	0.605	14.5	220
	20	−0.250	0.070	−6.3	0.305	0.625	15.7	130
	40	−0.300	0.020	−85	0.320	0.640	17.9	76
	80	−0.400	−0.080	−11.8	0.360	0.680	19.5	50

**Table 2:** Parameters used for the calculation of SERS enhancement factor (EF), for the peak at 1022 cm^−1^.

Nanomaterial	Specific Surface ^1^ (nm^2^/cm^2^)	Surface Coverage ^2^ (Molecules/cm^2^)	*N*^3^ (Molecules)	*I*_SERS_^4^ (Counts/Watt × s)	EF ^5^
**CuUWs**	1.2 × 10^15^	5.7 × 10^15^	2.5 × 10^8^	7.5 × 10^4^	0.7 × 10^4^
**AgNS@CuNW**	1.80 × 10^15^	8.2 × 10^15^	3.7 × 10^8^	25.0 × 10^4^	1.6 × 10^4^

^1^ Calculated area of the nanomaterial (in nm^2^) per unit section of exposed surface (in cm^2^). ^2^ Calculated by dividing column 1 by the surface area of one BT molecule (i.e., 0.22 nm^2^). ^3^ Calculated by multiplying column 3 by the laser spot area (4.5 × 10^−8^ cm^−2^). ^4^ Calculated by normalizing the peak area for the power and exposure time. ^5^ Calculated by Equation (3), using *I*_Raman_/*N*_Raman_ for BT reported in ref. [45].

## Data Availability

Data is available upon the reasonable request from the corresponding author.

## Supplementary Materials

The following are available online at https://www.mdpi.com/2079-4991/11/2/518/s1. Figure S1. Detail of SEM analysis of copper wires prepared in a PC template on GC working electrode; Figure S2. Detail of SEM analysis of copper wires prepared in an AAO template on Cu working electrode. Table S1. Analysis of the vibrational spectral features of benzenethiol and related literature references.
