# Supplementary material for: Surface Enhanced Raman Spectroscopy With Electrodeposited Copper Ultramicro-Wires With/Without Silver Nanostars Decoration
Source: Nanomaterials (Basel). 2021 Feb 18;11(2):518. doi: 10.3390/nano11020518 (PMC7922343; doi:10.3390/nano11020518)
Supplement: Supplementary file 1 [file nanomaterials-11-00518-s001.pdf]

Supplementary Material

Surface Enhanced Raman Spectroscopy with Electrodeposited Copper Ultramicro-Wires with/without Silver Nanostars Decoration

Margherita Longoni [1,2], Maria Sole Zalaffi [1], Lavinia de Ferri [3,4], Angela Maria Stortini [1], Giulio Pojana [3] and Paolo Ugo [1]*

[1] Department of Molecular Sciences and Nanosystems; University Ca' Foscari of Venice, via Torino 155, 30172 Venice, Italy. margherita.longoni@unimi.it (M.L.); mariasole.zalaffi@unive.it (M.S.Z.); stortini@unive.it (A.M.S.)
[2] Department of Chemistry; University of Milan, via C. Golgi 19, 20133 Milano, Italy
[3] Department of Philosophy and cultural Heritage; University Ca' Foscari of Venice, Dorsoduro 3484/d, 30123 Venice, Italy. lavinia.deferri@unive.it (L.d.F.); jp@unive.it (G.P.)
[4] Department of Collection Management-Museum of Cultural History; University of Oslo, Kabelgata 34, 0580, Oslo, Norway
* Correspondence: ugo@unive.it

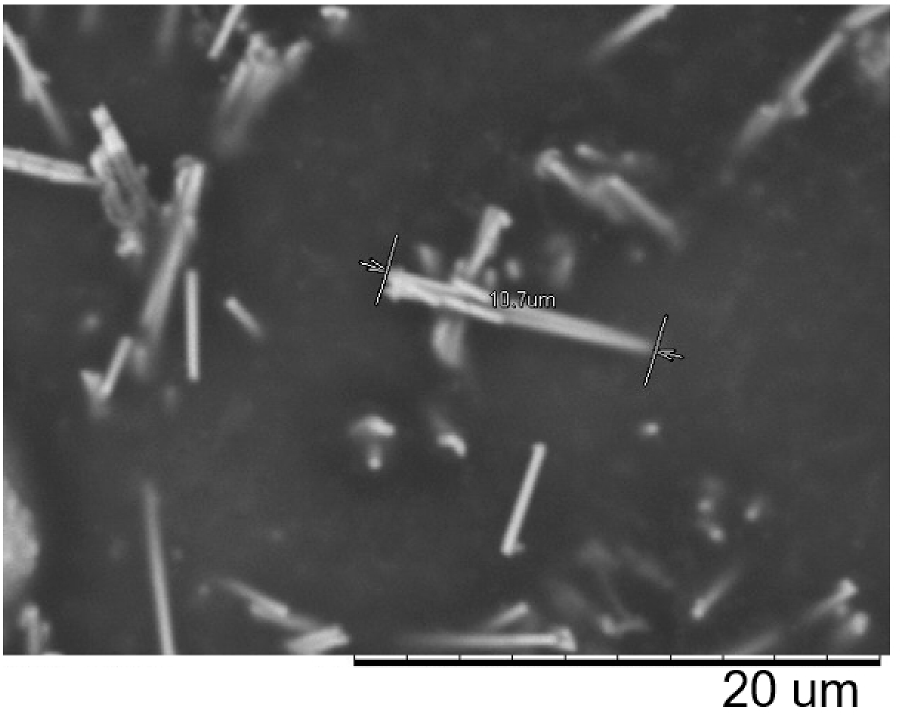

Figure S1. Detail of SEM analysis of copper wires prepared in PC template on GC working electrode. Other parameters as in Figure 7.

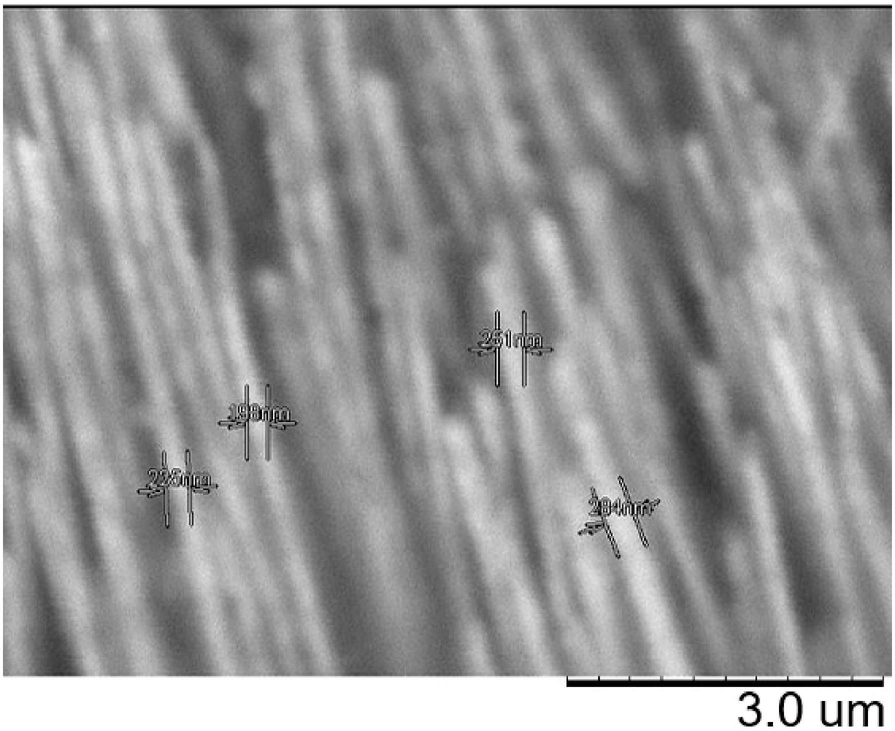

Figure S2. Detail of SEM analysis of copper wires prepared in AAO template on Cu working electrode. Other parameters as in Figure 8.

Table S1. Analysis of the vibrational spectral features of benzenethiol and related literature references.

Wavenumber (cm^{-1})	Assignation	Ref.
412-415	C-S stretching + ring in-plane deformation (β_{CCC})	[1–3]
466	Out of plane ring deformation	[4]
582	Ring in-plane deformation (β_{CCC}) + C-S stretching	[2]
688	Ring in-plane deformation (β_{CCC}) + C-S stretching	[1,2,5]
	γ_{CCC} (Out of plane bending)	[3]
994-998	Ring out-of-plane deformation + C-H out-of-plane bending	[1,6]
	C-H out-of-plane bending	[3,7]
	Ring in-plane deformation (β_{CCC})	[2]
	Ring breathing	[5]
1018-1025	Ring in-plane deformation + C-C symmetric stretching	[1,5,8]
	C-C symmetric stretching	[7]
	C-H in-plane deformation (β_{CH})	[2,3]
	Ring breathing	[5]
1070-1072	ring in plane deformation + C-S stretching	[2–5,8]
	C-C asymmetric stretching + C-S stretching	[1]
	C-C symmetric stretching	[7]
1570-1581	C-C stretching	[2,3,5,8]

Reference

1) Fontana, J.; Livenerec, J.; Bezares, F.J.; Caldwell, J. D.; Rendell, R.; Ratna, B.R. Large surface-enhanced Raman scattering from self-assembled gold nanosphere monolayers. *Appl. Phys. Lett.* **2013**, *102*, 201606, DOI: 10.1063/1.4807659.
2) Aggarwal, R. L.; Farrar, L. W.; Diebold, E. D.; Polla, D. L. Measurement of the absolute Raman scattering cross section of the 1584-cm−1 band of benzenethiol and the surface-enhanced Raman scattering cross section enhancement factor for femtosecond laser-nanostructured substrates. *J. Raman Spectrosc.* **2009**, *40*, 1331–1333, DOI: 10.1002/jrs.2396.

3) Joo, T. H.; Kim, M. S.; Kim, K. Surface-Enhanced Raman Scattering of Benzenethiol in Silver Sol. *J. Raman Spectrosc.*, *18*, 57-60, DOI: 10.1002/jrs.1250180111.
4) Gaetani, C.; Gheno, G.; Borroni, M.; De Wael, K.; Moretto, L.M.; Ugo, P. Nanoelectrode ensemble immunosensing for the electrochemical identification of ovalbumin in works of art. *Electrochim. Acta* **2019**, *312*, 72–79, DOI: 10.1016/j.electacta.2019.04.118.
5) Valley, N.; Greeneltch, N.; Van Duyne, R. P.; Schatz, G. C. A Look at the Origin and Magnitude of the Chemical Contribution to the Enhancement Mechanism of Surface-Enhanced Raman Spectroscopy (SERS): Theory and Experiment. *J. Phys. Chem. Lett.* **2013**, *4*, 2599−2604, DOI: 10.1021/jz4012383.
6) Habtamu, H. B.; Ugo, P. Miniaturized Enzymatic Biosensor via Biofunctionalization of the Insulator of Nanoelectrode Ensembles. *Electroanalysis* **2015**, *27*, 2187–2193, DOI: 10.1002/elan.201500115.
7) Park, S.; Lee, J.; Ko, H. Transparent and Flexible Surface-Enhanced Raman Scattering (SERS) Sensors Based on Gold Nanostar Arrays Embedded in Silicon Rubber Film. *ACS Appl. Mater. Interfaces* **2017**, *9*, 44088−44095, DOI: 10.1021/acsami.7b14022.
8) Dahlin, A. B. Size Matters: Problems and Advantages Associated with Highly Miniaturized Sensors. *Sensors* **2012**, *12*, 3018–3036, DOI: 10.3390/s120303018.
